# Antiretroviral therapy regimen modification rates and associated factors in a cohort of HIV/AIDS patients in Asmara, Eritrea: a 16-year retrospective analysis

**DOI:** 10.1038/s41598-023-30804-8

**Published:** 2023-03-14

**Authors:** Samuel Tekle Mengistu, Arsema Yohannes, Hermon Issaias, Mical Mesfn, Simon Zerufael, Aman Dirar, Habtemichael M. Teklemariam, Ghirmary Ghebrekidane Ghebremeskel, Oliver Okoth Achila, Saleem Basha

**Affiliations:** 1 Nakfa Hospital, Ministry of Health Northern Red Sea Branch, Nakfa, Eritrea; 2 Orotta College of Medicine and Health Sciences, Asmara, Eritrea; 3 Eritrean National Institute of Higher Education and Research, Mai-Nefhi College of Sciences, Mai-Nefhi, Eritrea; 4 Teseney Hospital, Ministry of Health Gash-Barka Branch, Teseney, Eritrea; 5 Unit of Clinical Laboratory Sciences, Orotta College of Medicine and Health Sciences, Asmara, Eritrea; 6 Unit of Allied Sciences, Orotta College of Medicine and Health Sciences, Asmara, Eritrea

**Keywords:** Epidemiology, HIV infections

## Abstract

Combined antiretroviral therapy (cART) durability and time to modification are important quality indicators in HIV/AIDs treatment programs. This analysis describes the incidence, patterns, and factors associated with cART modifications in HIV patients enrolled in four treatment centers in Asmara, Eritrea from 2005 to 2021. Retrospective cohort study combining data from 5020 [males, 1943 (38.7%) vs. females, 3077 (61.3%)] patients were utilized. Data on multiple demographic and clinical variables were abstracted from patient’s charts and cART program registry. Independent predictors of modification and time to specified events were evaluated using a multi-variable Cox-proportional hazards model and Kaplan–Meier analysis. The median (±IQR) age, CD4^+^ T-cell count, and proportion of patients with WHO Clinical stage III/IV were 48 (IQR 41–55) years; 160 (IQR 80–271) cells/µL; and 2667 (53.25%), respectively. The cumulative frequency of all cause cART modification was 3223 (64%): 2956 (58.8%) substitutions; 37 (0.7%) switches; and both, 230 (4.5%). Following 241,194 person-months (PMFU) of follow-up, incidence rate of cART substitution and switch were 12.3 (95% CI 11.9–12.8) per 1000 PMFU and 3.9 (95% CI 3.2–4.8) per 10,000 PMFU, respectively. Prominent reasons for cART substitution included toxicity/intolerance, drug-shortage, new drug availability, treatment failure, tuberculosis and pregnancy. The most common adverse event (AEs) associated with cART modification included lipodystrophy, anemia and peripheral neuropathy, among others. In the adjusted multivariate Cox regression model, Organisation (Hospital B: aHR = 1.293, 95% CI 1.162–1.439, *p* value < 0.001) (Hospital D: aHR = 1.799, 95% CI 1.571–2.060, *p* value < 0.001); Initial WHO clinical stage (Stage III: aHR = 1.116, 95% CI 1.116–1.220, *p* value < 0.001); NRTI backbone (D4T-based: aHR = 1.849, 95% CI 1.449–2.360, *p* value < 0.001) were associated with increased cumulative hazard of treatment modification. Baseline weight (aHR = 0.996, 95% CI 0.993–0.999, *p* value = 0.013); address within Maekel (aHR = 0.854, 95% CI 0.774–0.942, *p* value = 0.002); AZT-based backbones (aHR = 0.654, 95% CI 0.515–0.830, *p* value < 0.001); TDF-based backbones: aHR = 0.068, 95% CI 0.051–0.091, *p* value < 0.001), NVP-based anchors (aHR = 0.889, 95% CI 0.806–0.980, *p* value = 0.018) were associated with lower cumulative hazards of attrition. The minimal number of switching suggests inadequate VL testing. However, the large number of toxicity/intolerance and drug-shortage driven substitutions highlight important problems in this setting. Consequently, the need to advocate for both sustainable access to safer ARVs in SSA and improvements in local supply chains is warranted.

## Introduction

The 2021 Joint United Nations Program on HIV/AIDS (UNAIDS) fact sheet estimated that in 2020, there were 37.7 million [30.2 million–45.1 million] people living with Human Immunodeficiency Virus (HIV) including 20.6 million [16.8 million–24.4 million] in Eastern and Southern Africa (ESA)^1^. Fundamentally, the report suggests that more than half the global population of people living with HIV/AIDS (PLWHA) are found in ESA and that the region has the highest incidence of AIDS-related mortality, 310,000 [220,000–470,000]. Fortunately, the report notes that combination antiretroviral therapy (cART) coverage in the region has greatly improved and that currently 16.0 million [15.4 million–16.1 million] or 77% [60–92%] are accessing treatment^1^.

The benefits associated with improvements in cART are adequately described in existing literature and these include sustained virus suppression, immune reconstitution/recovery, and dramatic reductions in HIV/AIDS morbidity and mortality. Low viremia is also associated with reduced HIV transmission rates^2^. Despite these positives and the decade-long successes in improvements of cART coverage in Low and Middle Income Countries (LMICs); treatment programs in the region are still facing multiple challenges. These include limited access to experts and relevant diagnostics (HIV/RNA viral load (HIV/RNA-VL), clinical chemistry, and genotype testing). More importantly, the use of second-generation Non-Nucleoside inhibitors (NNRTIs), Integrase inhibitors [INSTIs], fixed-dose cART formulations without older Nucleoside Reverse Transcriptase Inhibitor (NRTI) backbones, or newer, less toxic drug classes like Entry inhibitors (EIs) are still not part of the standard of care. Instead, cART availability in most Sub-Saharan Africa (SSA) countries is restricted to a limited formulary comprising of a small-number of well-studied combination of old anti-retroviral (ARVs). This development is largely attributable to the World Health Organization (WHO) proposition that countries in the region should adopt a public health based approach to cART [WHO 2006]^3^.

Due to the limited number of options, particularly anchor drugs; maximization of first or second line regimen durability is recognized as a key programmatic priority^4,5^. At present, the most straight forward pathway to maximization of cART durability involves the adoption of prudent modification strategies—decreasing substitution rates and limiting unnecessary switching to second-line regimens. Multiple studies have confirmed drug modifications in cART care settings can be prompted by a number of medication and patients-dependent factors. The former includes the need to limit adverse events (AEs); treatment failure; new drugs, eliminate drug—drug interactions (DDIs); reduce pill burden or dosing frequency; and availability of new drugs. On the other hand, there are many well-recognized concerns regarding inappropriate modification of cART. Possible risks include the development of viral cross-resistance to specific ARVs^6^ and increased mortality^7^. This may compromise the preservation of these medications as components of alternative first-line cART regimens and/or second line options^8^. Importantly, data suggests that subsequent regimens have progressively shorter durability and are costlie^9^. These, it has been suggested; are important concerns in SSA where cART options are severely limited^6^.

Altogether, we can argue that despite the necessary occurrence of treatment modifications in the lifelong course of cART; frequency, patterns and correlates of treatment modifications (particularly in the pre- and post-INSTI era) are poorly characterized in SSA. Therefore, the primary objective of this study was to describe incidence, patterns, and factors associated with drug modifications in patients on cART in multiple treatment centers in Asmara, Eritrea. This information can be leveraged to develop preventive interventions aimed at enhancing durability of the first regimen.

## Methodology

### Study design and setting

We conducted a retrospective cohort study at four cART centers in Asmara, Eritrea. These hospitals include: Orotta National Referral Hospital (ONRH); Halibet National Referral hospital (HRH); Sembel hospital (SH); and Haz-Haz Zonal Referral Hospital (HzH) located in Asmara, the capital city of Eritrea. The central zone is the largest administrative region out of the six zones in the country. Before the decentralization of services to other zones in 2010; these were the only institutions that were offering ART to people living with HIV in the country. In total, around 6235 adults have accessed cART in these clinics since the inception of the program in 2005. Treatment is guided by the Eritrean National Adult guidelines developed by Eritrean Ministry of Health (EMoH)^10^. Treatment eligibility has evolved tandem with WHO recommendations—CD4^+^ T-cell count of ≤ 200 cells/μL; ≤ 350 cells/μL; ≤ 500 cells/μL, and test-and-treat approach in 2005, 2010, 2015, and 2016, respectively.

Drug combinations are designed as per WHO recommendations around the following ARVs: Nucleotide reverse transcriptase inhibitors (NRTI) (Zidovudine (ZDV), Lamivudine (3TC), Emtricitabine (FTC), Tenofovir disoproxil fumarate (TDF), Didanosine (DDI), Abacavir (ABC)—Stavudine (d4T); Non-nucleoside reverse transcriptase inhibitors (NNRTIs): Nevirapine (NVP), Efavirenz (EFV); Protease inhibitors (PIs): Lopinavir/ritonavir (LPV/r); and Intergrase inhibitors (INSTIs) (Dolutegravir (DTG). Major guidelines changes were adopted in 2010. Integrase Inhibitors (DTG in particular) was not readily available before 2020. These drugs along with required laboratory services are provided *gratis*. Dispensing of cART occurs after every three months or as per scheduled clinical visits. During these visits, additional care (drug adherence counseling, monitoring of toxicity, treatment of opportunistic infections (OIs), among others) is provided.

### Study cohort description

**Figure 1:**
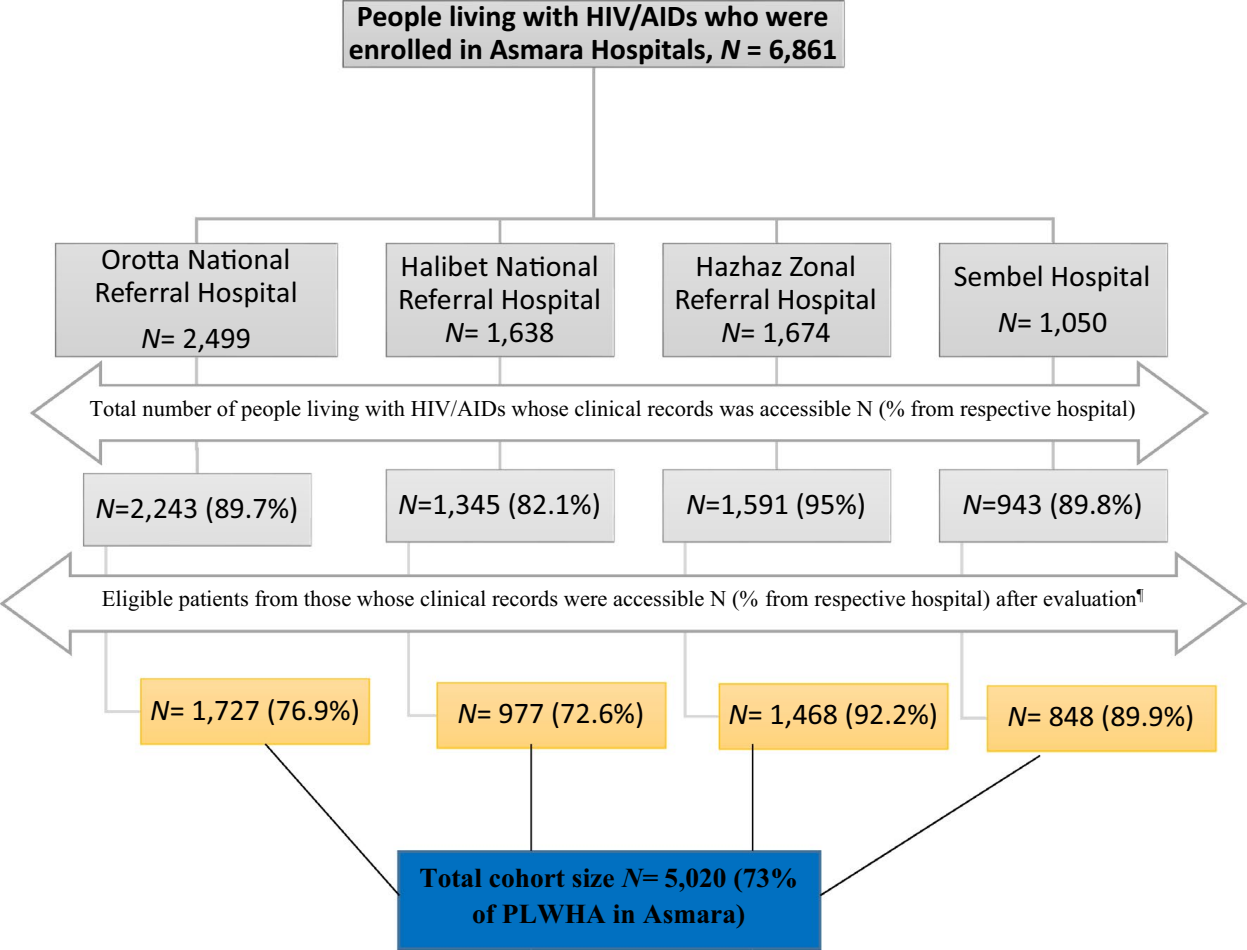
Flow diagram for study participants’ recruitment in PLWHA in Asmara Hospitals, 2005–2021.

All adult patients with HIV/AIDS who attended the four outpatient HIV follow-up clinics from 2005–2021 were enrolled in the study. All patients included in the analysis were initiated on a variety of cART backbones (AZT/3TC, D4T/3TC, ABC/3TC; TDF/3TC, TDF/FTC, ABC/DDI, ABC/3TC/AZT). Anchor drugs include ATV/r, DTG, EFV, LPV/r, and NVP. Excluded individuals were: those whose records were repeated in another hospital, incomplete data of cART regimen modification and/or illegible. As per the standard care at the HIV clinics, all the patients had their HIV-1 infection status confirmed positive by nucleic acid based testing (Real time Polymerase Chain Reactions (PCR) TaqMan1 HIV-1 Test). See Fig. 1.

### Data collection procedure

De-identified and anonymous patient data were obtained from the cART registries of the respective hospitals and patient cards. Accordingly, all clinical cards were reviewed for demographic information (Sex, age, address, occupation, level of education, marital status); clinical (baseline weight, initial WHO clinical stage), and laboratory data (initial CD4^+^ T-cell count cells/mm^3^). Regimen data assessed included First-line cART combination, subsequent modifications (substitutions and/or switching) and reasons for cART modifications. The process was undertaken by trained health professionals and was closely monitored by the principal investigator and supervisors. Data collection was carried out in September-December 2021.

### Operational definition and study outcome

The primary outcome of this study was to determine the factors associated with cART modification. This was evaluated between the first documented time of cART initiation (baseline) and the last time occurring within the end of the study period (December 31, 2021). In the process, the following operational definitions were used:

- *Drug substitution*: this was defined as class (NRTI or NNRTI) for class (NRTI or NNRTI) replacement of one or more First-line cART regimen^6^. Due to the fact that 3TC and FTC are therapeutically interchangeable, a change between the two drugs was not regarded as a substitution.
- *Drug Switching*: Drug switch was defined as a change from the first-line to Second line regimen.

### Statistical analysis

Analysis was conducted using IBM SPSS version 26 (SPSS Inc., Version 26.0, Chicago, IL, USA) and Stata version 14.0 (Stata Corporation, College Station, Texas, USA). Descriptive statistics for categorical variables were analyzed using chi-square (χ^2^)/Fishers exact test and summarized using counts (frequency) and proportions (percentages). Normality test was conducted prior to any statistical computation and quantitative data was summarized using median with Interquartile range (IQR). The incidence rate of drug modification was calculated by dividing the number of patients with initial first-line ART modification by the total number of persons-years of follow up. Furthermore, Kaplan–Meier estimates and log-rank tests were carried out to compare the cumulative incidence of cART modification between different categories of patient specific characteristics. Finally, a Cox regression hazard was performed to identify the predictors of therapy modification. The variables that were considered in a multivariate-adjusted Cox proportional hazards model of drug modification were age at treatment initiation, gender, baseline functional status, baseline clinical status, baseline CD4^+^ count and baseline body weight. A backward-selection procedure was used to create these adjusted models, with a variable being included in the model if it resulted in an improvement in the model fit. The final results are presented as AHR with a 95% confidence interval (CI). In all instances, *p* value < 0.05 was regarded as significant.

### Ethical considerations

Ethical approval for the study and experimental protocols used was obtained from Eritrean Ministry of Health (MOH) research ethical committee (Letter of reference: # 08/2021) and communicable disease control (CDC). All the information gathered was De-identified, held with utmost confidential responsibility and used only for this study’s purpose.As the study also included data based on patients’ clinical card records, consent for the data access was waived by the ethical committee in place of the patients. This study conforms to the principles outlined in the Declaration of Helsinki.

### Ethical approval and consent

Ethical clearance was obtained from the Health Research Ethics and Protocol Review Committee of the Ministry of Health. Consent to participate was not obtained from the patients. All ethical and professional consideration were followed during the study to make patients identity strictly confidential.

## Results

### Baseline clinical and demographic characteristics of study population

**Figure 2:**
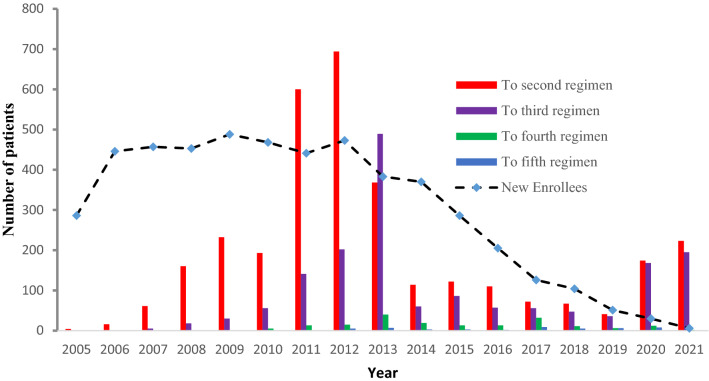
Continuous field information on enrollment and modification frequencies.

A total of 5020 patients [Hospital A: 977 (19.5%), Hospital B: 1468 (29.2%), Hospital C: 1727 (34.4%), And Hospital D: 848 (16.9%)] with 5.8 (IQR 3.4–9.4) years of follow-up time enrolled in the study. The median (± IQR) age at cART initiation was 48 (IQR 41–55) years and participation favored females, 3077 (61.3%). Majority of participants were from central zone 4219 (84%), married 2262 (45.1%) and had secondary level education 2109 (42%) while nearly half of patients were employed 2221 (44.2%). In terms of disease clinical conditions, 2667 (53.25%) were either in WHO stage III or IV. Moreover, the median (± IQR) baseline CD4^+^ cell count was 160 (IQR 80–271) cells/µL with a significant proportion of participants, 3083 (61.3%), having CD4^+^ T-count < 200 cells/mm^3^. See Fig. 2 and Table 1.

**Table 1:** Baseline Sociodemographic and clinical characteristics of PLWHA in hospitals of Asmara per cART regimen modification types, 2005–2021.

Patients’ characteristics	Total N (%)	No change	Substitution	Switch	Both	*p* value
	5020	1797 (35.8)	2956 (58.8)	37 (0.7)	230 (4.5)	
**Organization care**						
A	977 (19.5)	262 (14.6)	674 (22.8)	5 (13.5)	36 (15.7)	**< 0.001 (136.03)**
B	1468 (29.2)	617 (34.3)	768 (26)	7 (18.9)	76 (33)	
C	1727 (34.4)	528 (29.4)	1090 (36.9)	15 (40.5)	94 (40.9)	
D	848 (16.9)	390 (21.7)	424 (14.3)	10 (27)	24 (10.4)	
**Gender**						
Female	3077 (61.3)	1127 (62.7)	1801 (60.9)	23 (62.2)	126 (54.8)	0.121 (5.82)
Male	1943 (38.7)	670 (37.3)	1155 (39.1)	14 (37.8)	104 (45.2)	
**Age in years, median (IQR)**	48 (41–55)	47 (39–53)	49 (43–55)	41 (34–51)	48 (41–55)	**< 0.001**^a^
16–30	337 (6.7)	161 (9)	149 (5)	8 (21.6)	26 (11.3)	**< 0.001 (91.08)**
31–45	1574 (31.4)	654 (36.4)	843 (28.5)	15 (40.5)	58 (25.2)	
46–60	2540 (50.6)	800 (44.5)	1608 (54.4)	12 (32.4)	119 (51.7)	
> 60	569 (11.3)	182 (10.1)	356 (12)	2 (5.4)	27 (11.7)	
**Address**						
Central zone	4219 (84)	1469 (81.7)	2513 (85)	31 (83.8)	206 (89.6)	**0.002 (14.37)**
Outside central zone	801 (16)	328 (18.3)	443 (15)	6 (16.2)	24 (10.4)	
**Occupation**						
Unemployed	2799 (55.8)	1050 (58.4)	1593 (53.9)	23 (62.2)	132 (57.4)	**0.018 (10.1)**
Employed	2221 (44.2)	747 (41.6)	1362 (46.1)	14 (37.8)	98 (42.6)	
**Education Level**						
Illiterate	357 (7.1)	138 (7.7)	199 (6.7)	3 (8.1)	17 (7.4)	0.9 (6.28)
Primary	971 (19.3)	352 (19.6)	571 (19.3)	7 (18.9)	41 (17.8)	
Middle	1274 (25.4)	447 (24.9)	752 (25.4)	12 (32.4)	63 (21.7)	
Secondary	2109 (42.0)	756 (42.1)	1243 (42.1)	15 (40.5)	95 (41.3)	
Higher	309 (6.2)	104 (5.8)	191 (6.5)	0 (0.0)	14 (6.1)	
**Marital status**						
Single	1146 (22.8)	458 (25.5)	609 (20.6)	11 (29.7)	68 (29.6)	**< 0.001 (42.09)**
Married	2262 (45.1)	771 (42.9)	1368 (46.3)	1368 (46.3)	104 (45.2)	
Widowed	946 (18.8)	298 (16.6)	611 (20.7)	611 (20.7)	34 (14.8)	
Divorced	66 (13.3)	270 (15)	368 (12.4)	368 (12.4)	24 (10.4)	
**Initial weight in Kg, median (IQR)**	50.0 (43.5–58)	50.0 (43.0–58.45)	50 (44–58)	49 (38.5–55.75)	50.0 (43–55.6)	0.366^a^
< 45	1611 (32.11)	610 (33.9)	910 (30.8)	15 (40.5)	76 (33)	**0.031 (13.89)**
45–55	1777 (35.4)	601 (33.4)	1068 (36.1)	12 (32.7)	96 (41.7)	
> 55	1632 (32.5)	586 (32.6)	978 (33.1)	10 (27)	58 (25.2)	
**Initial clinical stage**						
I	1434 (28.6)	560 (31.2)	803 (27.2)	9 (24.3)	62 (27)	**< 0.001 (38)**
II	919 (18.3)	290 (16.1)	564 (19.1)	9 (24.3)	56 (24.3)	
III	2132 (42.5)	727 (40.5)	1305 (44.1)	10 (27)	90 (39.1)	
IV	535 (10.7)	220 (41.1)	284 (53.1)	9 (24.3)	22 (9.6)	
**Initial CD4 count in cells/mm**^**3**^, **median (IQR)**	160 (80–271)	172 (80–314)	158.5 (82–248)	113 (66–309)	140 (65–213.5)	**< 0.001**^**a**^
1–100	1582 (31.5)	562 (31.3)	913 (30.9)	16 (43.2)	91 (39.6)	**< 0.001 (106.44)**
101–200	1491 (29.7)	443 (24.7)	967 (32.7)	8 (21.6)	73 (31.7)	
201–350	1240 (24.7)	434 (24.2)	757 (25.6)	7 (18.9)	42 (18.3)	
> 350	707 (14.1)	358 (19.9)	319 (10.8)	6 (16.2)	24 (10.4)	
**Initial functional status**						
Bedridden	210 (4.2)	65 (3.6)	133 (4.5)	2 (5.4)	10 (4.3)	**< 0.001 (33.1)**
Ambulatory	1034 (20.6)	448 (24.9)	538 (18.2)	6 (16.2)	42 (18.3)	
Work	3776 (75.2)	1284 (71.5)	2285 (77.3)	29 (78.4)	178 (77.4)	
**NRTI**						
ABC/3TC	104 (2.1)	19 (18.3)	80 (76.9)	1 (1.0)	4 (3.8)	**< 0.001 (2668.14)**
AZT/3TC	1757 (35)	337 (19.2)	1312 (74.7)	21 (1.2)	87 (5)	
D4T/3TC	1761 (35.1)	164 (9.3)	1472 (83.6)	5 (0.3)	120 (6.8)	
TDF/FTC	1399 (27.8)	1277 (91.3)	92 (6.6)	1 (0.7)	19 (1.4)	
**NNRTI**						
EFV	2425 (48.3)	1456 (81)	887 (30)	21 (56.8)	61 (26.5)	**< 0.001 (1234.08)**
NVP	2586 (51.5)	333 (18.5)	2068 (69.9)	16 (43.2)	169 (73.5)	
None	9 (0.2)	8 (0.5)	1 (0.1)	0 (0.0)	0 (0.0)	

Significant values are in bold.

TDF, Tenofovir disoproxil fumarate; FTC, Emtricitabine; AZT, Zidovudine (AZT); 3TC, Lamivudine; ABC, Abacavir; D4T, Stavudine; NVP, Nevirapine; EFV, Efavirenz. NNRTI, non-nucleoside reverse transcriptase inhibitor; NRTI, nucleoside reverse transcriptase inhibitor.

^a^Kruskal-Wallis test-1 way ANOVA test.

**Figure 3:**
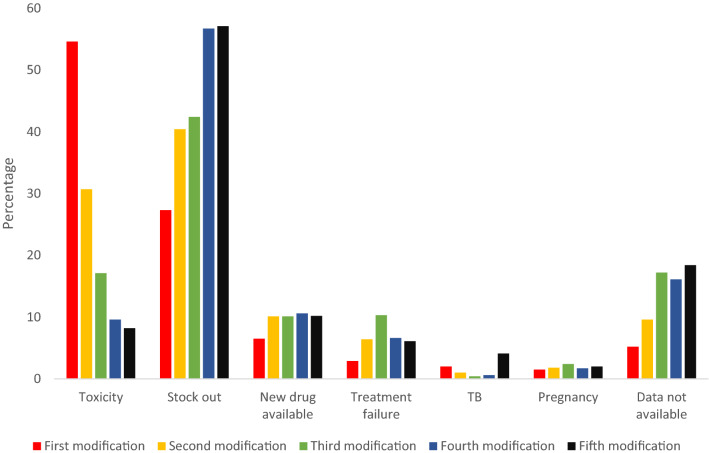
Distribution of documented reasons for cART regimen modification among PLWHA in hospitals of Asmara, 2005–2021. The percentages represent the proportion of the documented reason of modification within the order of the modification.

**Figure 4:**
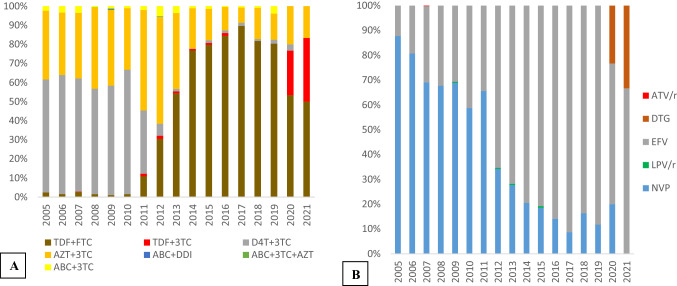
Distribution of cART regimens’ backbones and anchor drugs prescribed across study years among PLWHA in hospitals of Asmara, 2005–2021. Distribution of (**A**) backbone and (**B**) anchor drug classes prescribed in first cART regimens by year (2005‒2021). Anchor drugs: NNRTI Non-nucleoside reverse transcriptase inhibitor, PI Protease inhibitor (LPV/r: Lopinavir/ritonavir and ATV/r: Atazanavir/ritonavir), INSTI intergrase strand transfer inhibitor (DTG: Dolutegravir). Backbone drugs: ABC Abacavir, TDF Tenofovir disoproxil fumarate; 3TC: lamivudine, FTC: emtricitabine.

**Figure 5:**
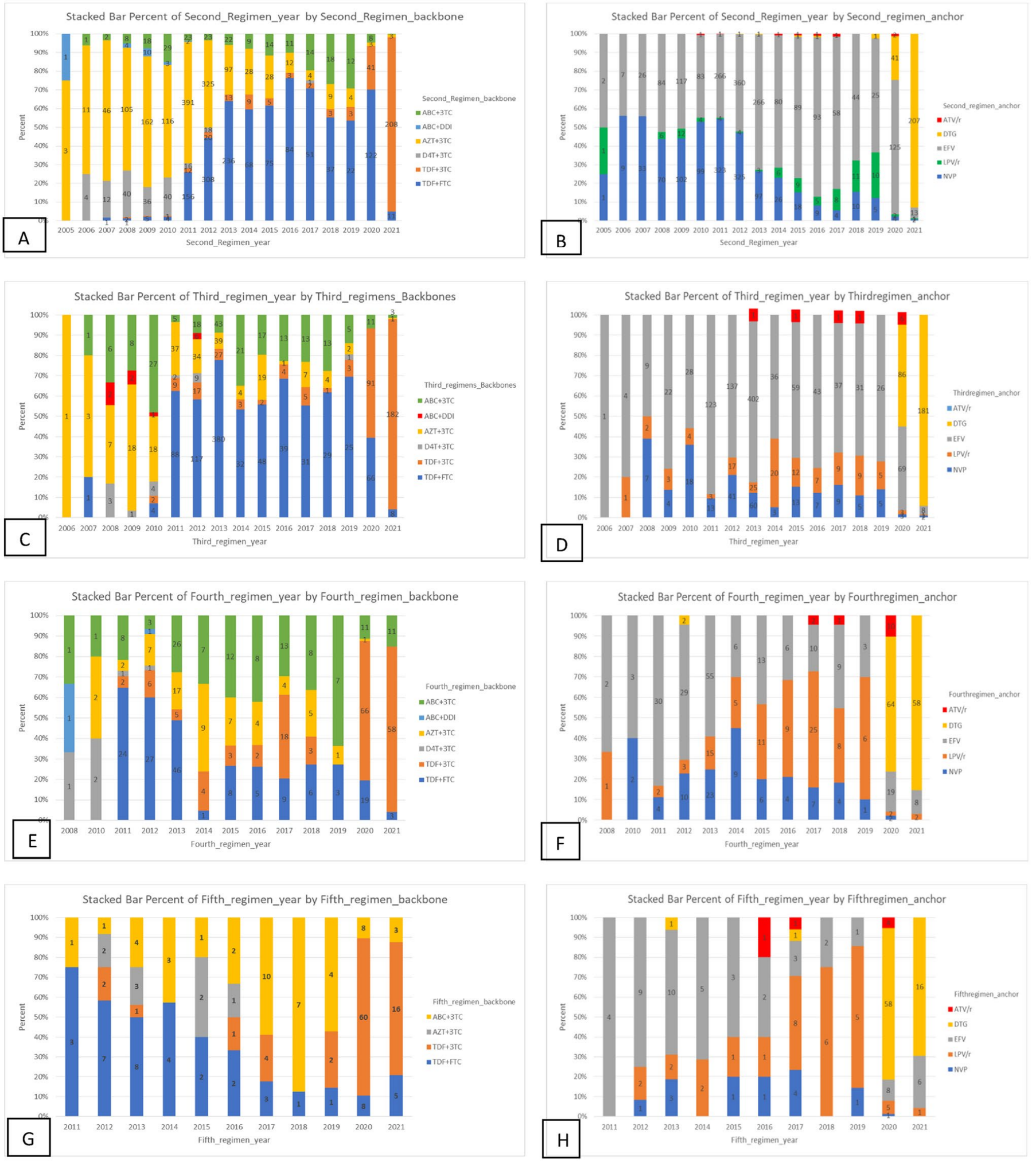
Distribution of cART regimens’ backbones and anchor drugs that were substituted and switched from 1st up to 5th regimen across study years among PLWHA in hospitals of Asmara, 2005–2021. Distribution of (**A**,**C**,**E**,**G**) backbone and (**B**,**D**,**F**,**H**) anchor drug classes prescribed in first cART regimens by year (2005‒2021). Anchor drugs: NNRTI Non-nucleoside reverse transcriptase inhibitor, PI Protease inhibitor (LPV/r: Lopinavir/ritonavir and ATV/r: Atazanavir/ritonavir), INSTI intergrase strand transfer inhibitor (DTG: Dolutegravir). Backbone drugs: ABC Abacavir, TDF Tenofovir disoproxil fumarate; 3TC: lamivudine, FTC: emtricitabine.

Prior to the WHO consolidated guidelines updates of 2010, the most frequently prescribed initial regimen was d4T/3TC/NVP or EFV (35.1%) and AZT/3TC/NVP or EFV (35%) (Fig. 3). From 2011 to 2021, the most frequently prescribed initial regimen was TDF/FTC/ NVP or EFV (27.8%). See Figs. 4 and 5.

### Prevalence of cART substitution and switch, and associated factors

Of the 2956 (58.8%, 95% CI 57.5–60.2) patients who incurred a drug substitution at least once, 1560 (52.5%) involved a single NRTI substitution, 310 (10.43%) involved a single NNRTI substitution, and 1075 (36.2%) involved both an NRTI and an NNRTI substitution. Further, 230 (4.5%, 95% CI 4–5.1) patients experienced both types of cART modification (drug substitution and drug switch) while 37 (0.7%, 95% CI 0.5–0.9) patients switched from NNRTI-based to PI-based regimen. Hospital C had significantly higher proportion of drug substitution 1090 (36.9%) and switching 15 (40.5%) or both 94 (40.9%) patients. High record of cART switching was associated with lower median age [41, IQR 34–51 years]; *p* value < 0.001, Kruskal–Wallis test, and CD4^+^ T-cell count [113, IQR 66–309 cell/µL]; *p* value < 0.001, Kruskal–Wallis test. Compared to patients Outside the central zone, patients from central zone had higher record of substitution, switching or both, 2513 (85%), 31 (83.8%) and 206 (89.6%), *p* value = 0.002. Further, median (± IQR) CD4^+^ T-cell count was associated with substitution, switching or both—158.5 cells/mm^3^ (82–248 cells/mm^3^), 113 cells/mm^3^ (66–309 cells/mm^3^), 140 cells/mm^3^ (65–213.5 cells/mm^3^), Kruskal–Wallis test *p* value < 0.001. Concerning cART regimen, ABC/3TC, AZT/3TC, D4T/3TC were associated with higher proportions of substitution (80 (76.9%) vs. 1312 (74.7%) vs 1472 (83.6%), respectively) compared to TDF/FTC based back bones (92 (6.6%). Similarly, EFV was associated with lower substitution, 887 (30%) vs. 2068 (69.9%) for NVP. Additional factors that were associated with substitution, switching or both included occupation, marital status, baseline weight in Kg, baseline WHO clinical stage, and baseline functional status. See Table 1 for more details.

### Documented reasons for cART Regimen modification and distribution of cART regimens prescribed across study years

Figure 3 presents documented reasons for cART modification. In order of decreasing frequency, the most common documented reason for first-time regimen change included toxicity, 1759 (54.6%); Stock out, 879 (27.3%); new drug availability, 209 (6.5%); treatment failure, 94 (2.9%); Tuberculosis (TB), 66 (2.0%), and pregnancy 48 (1.5%). Predominant reasons for second time regimen change included stock out, 658 (40.4%); toxicity (497(30.7%); new drug availability, 165 (10.1%); treatment failure, 104 (6.4%); pregnancy, 30 (1.8%), and TB (16 (1.0%)). For the third modification, the most common reason for change was stock out, 210 (57.1%); toxicity, 85 (17.1%); treatment failure, 51 (10.3%); new drug, 50 (10.1%); pregnancy, 12 (2.4%) and TB, 2 (0.4%). Similarly, fourth change was associated with Stock out, 102 (56.7%); new drug availability, 19 (10.6%); toxicity, 17 (9.6%); treatment failure, 12 (6.6%) and TB, 1 (0.6). Lastly, fifth drug modifications were due to Stock outs, 28 (57.1%); new drug availability, 5 (10.2%); toxicity, 4 (8.2), and treatment failure (3 (6.1%)). The most common adverse event in the early-NNRTIs based cART backbone in the 1^st^-5^th^ modification events were lipodystrophy, 481 (27.36%); anemia, 144 (8.19%); peripheral neuropathy, 96 (5.46%) among others. In the NRTIs group, adverse events observed included lipodystrophy, 481 (27.35%); Anemia, 144 (8.19%); peripheral neuropathy, 96 (5.46%), and skin rash, 72 (4.09%).

### Distribution of anchor drug class and backbone drugs in cART regimens by year

The most prescribed cART backbones at the start of the programme in 2005 were d4T/3TC, 169 (59.1%), and AZT/3TC, 103 (36%). The dominant anchor drug in the same period was NVP, 251 (87.8%). Treatment initiations on d4T/3TC cART backbone persisted until 2011 and dropped drastically, in 2012 to 30 (6.36%). In 2011, the dominant cART backbone was AZT/3TC, 232 (52.6%). Treatment initiation on TDF/FTC cART increased from a low point of 48 (10.9%) in 2011 to a peak of 113 (89.7%) before declining to 41 (50.3%) in 2021. The use of EFV as an anchor drug at cART initiation increased gradually from 35 (12.2%) in 2005 to 45 (88.2%) in 2019. DTG usage was initiated in 2020. See Fig. 5 for additional information on 2-5^th^ substitutions.

### Kaplan–Meier Survival analysis of cART substitution and switch rates

Following 241,194 person-months of follow-up (PMFU), incidence rate of cART substitution and switch was 12.3 (95% CI 11.9–12.8) per 1000 PMFU and 3.9 (95% CI 3.2–4.8) per 10,000 PMFU, respectively. Kaplan–Meier survival analysis for cART substitution and switch was then conducted to compare the mean duration of survival among sub-groups of patient characteristics. In this analysis, patients followed in Hospital A had significantly shorter mean duration to drug substitution [63 (95% CI 60–67) months; log rank *p* value < 0.001]. Moreover; patients aged > 60 years were observed to have significantly shorter mean duration to substitution [62 (95% CI 57–67) per 1000 PMFU while Patients from central zone and weight < 45 kg had shorter mean survival duration [68 (95% CI 66–70) months log rank *p* value = 0.02 and 65 (95% CI 62–68) months, log rank *p* value = 0.004 respectively]. WHO clinical stage 3 and 4, CD4^+^ T-cell count < 200 cells/µL and bed ridden patients were also observed to have shorter mean duration to drug substitution. Regarding ART regimen, TDF/FTC backbone and NVP from the NNRTI showed significantly longer mean duration to substitution. Concerning cART switching, patients followed in Hospital A had longer mean duration to ART switch of 185 (95% CI 183–187) months as compared to Hospital B (177, 95%CI: 173–182) months, Hospital C (182, 95% CI 178–185) months and Hospital D (178, 95% CI 173–183) months; log rank *p* value = 0.002. See Table 2 for details.

**Table 2:** cART regimen substitution and switch rates, Kaplan–Meier survival estimates and associated factors among PLWHA in hospitals of Asmara, 2005–2021.

Cohort characteristics	Substitution rate of initial regimen in 1000 person months (95%CI)	Mean regimen retention duration in months, 95% CI	*p* value (log-rank)	Switch rate in 10,000 person months, (95%CI)	Mean cART First-line retention in months, 95% CI	*p* value (log-rank)
**Total**	12.3 (11.9–12.8)	69 (67–70)		3.9 (3.2–4.8)	185 (182–187)	
**Organization unit**						
A	14.1 (13.1–15.3)	63 (60–67)	**< 0.001 (34.3)**	1.4 (0.6–3)	185 (183–187)	**0.002 (14.6)**
B	12.8 (12–13.80	65 (62–69)		2.6 (1.5–4.2)	177 (173–182)	
C	11.6 (10.9–12.3)	74 (72–77)		5.6 (4.3–7.4)	182 (178–185)	
D	11.1 (10–12.2)	69 (64–73)		4.9 (3.1–7.8)	178 (173–183)	
**Gender**						
Female	12.3 (11.8–12.9)	68 (66–71)	0.7 (0.1)	3.3 (2.5–4.4)	185 (182–189)	0.09 (2.8)
Male	12.3 (1.7–13.1)	69 (66–72)		4.8 (3.6–6.4)	176 (173–179)	
**Age in years**						
16–30	9.4 (8.1–11)	78 (72–85)	**< 0.001 (30.5)**	6.4 (3.5–11.5)	148 (139–157)	0.2 (3.3)
31–45	11.1 (10.4–11.9)	73 (70–76)		4.3 (3.1–6.1)	181 (176–186)	
46–60	13.1 (12.5–13.8)	66 (64–68)		3.4 (2.5–4.7)	183 (180–186)	
> 60	14.1 (12.7–15.6)	62 (57–67)		3.1 (1.5–6.2)	188 (183–193)	
**Address**						
Maekel	12.6 (12.1–13.1)	68 (66–70)	**0.02 (4.7)**	4 (3.2–5)	185 (182–187)	0.5 (0.4)
Outside Maekel	11.2 (10.2–12.2)	73 (68–77)		3.3 (1.9–5.6)	175 (170–181)	
**Initial weight**						
< 45	13.1 (12.3–14)	65 (62–68)	**0.004 (10.8)**	4.3 (3–6.2)	176 (173–179)	0.1 (3.8)
45–55	12.6 (11.9–13.4)	68 (65–71)		4.5 (3.3–6.2)	180 (175–185)	
> 55	11.4 (10.7–12.1)	72 (69–75)		2.9 (1.9–4.3)	187 (184–191)	
**Initial WHO Clinical stage**						
Stage 1	10.7 (10–11.5)	75 (72–78)	**< 0.001 (29.8)**	3.4 (2.3–5)	184 (179–189)	0.1 (6.1)
Stage 2	12.2 (11.3–13.2)	70 (66–73)		4.5 (3–6.9)	174 (169–180)	
Stage 3	13.5 (12.8–14.2)	65 (62–67)		3.4 (2.4–4.7)	186 (184–189)	
Stage 4	13.2 (11.7–14.8)	65 (59–70)		6.5 (3.8–11)	172 (163–181)	
**Initial CD4 count**						
1–100	13.9 (13–14.8)	63 (60–66)	**< 0.001 (95.2)**	5 (3.6–7)	181 (178–185)	0.07 (6.7)
101–200	13.7 (12.9–14.6)	65 (62–68)		4.3 (3–6.1)	184 (180–189)	
201–350	12.1 (11.3–13)	69 (66–72)		2.5 (1.5–4.1)	171 (167–175)	
> 350	7.6 (6.8–8.5)	88 (83–93)		3.4 (2–5.8)	180 (174–187)	
**Initial functional status**						
Bedridden	17.1 (14.4–20.3)	53 (45–60)	**< 0.001 (36.2)**	5.2 (1.9–14)	170 (164–175)	0.6 (0.7)
Ambulatory	13.5 (12.4–14.7)	62 (58–65)		3.5 (2.1–5.9)	173 (168–178)	
Work	11.9 (11.4–12.4)	71 (69–73)		3.9 (3.1–4.9)	185 (182–187)	
**NRTI**						
ABC-Based	18.7 (15–23.2)	52 (41–62)	**< 0.001 (1940)**	6.8 (2.2–21.2)	167 (156–177)	0.2 (4.5)
AZT-Based	12.7 (12.1–13.5)	69 (67–72)		5.2 (4–6.9)	184 (181–187)	
D4T-Based	26.1 (24.8–27.5)	36 (35–37)		2.9 (1.8–4.6)	161 (152–171)	
TDF-Based	1 (0.8–1.3)	167 (159–176)		2.8 (1.8–4.2)	179 (170–189)	
**NNRTI**						
EFV-Based	6.7 (6.3–7.2)	94 (91–97)	**< 0.001 (688)**	3.8 (2.9–5)	182 (179–185)	0.6 (0.1)
NVP-Based	18.6 (17.8–19.4)	50 (48–52)		4 (3–5.4)	184 (180–187)	

Significant values are in bold.

TDF, Tenofovir disoproxil fumarate; FTC, Emtricitabine; AZT, Zidovudine (AZT); 3TC, Lamivudine; ABC, Abacavir; D4T, Stavudine; NVP, Nevirapine; EFV, Efavirenz. NNRTI, non-nucleoside reverse transcriptase inhibitor; NRTI, nucleoside reverse transcriptase inhibitor.

### cART modification rates and mean survival duration at different intervals of follow up

**Figure 6:**
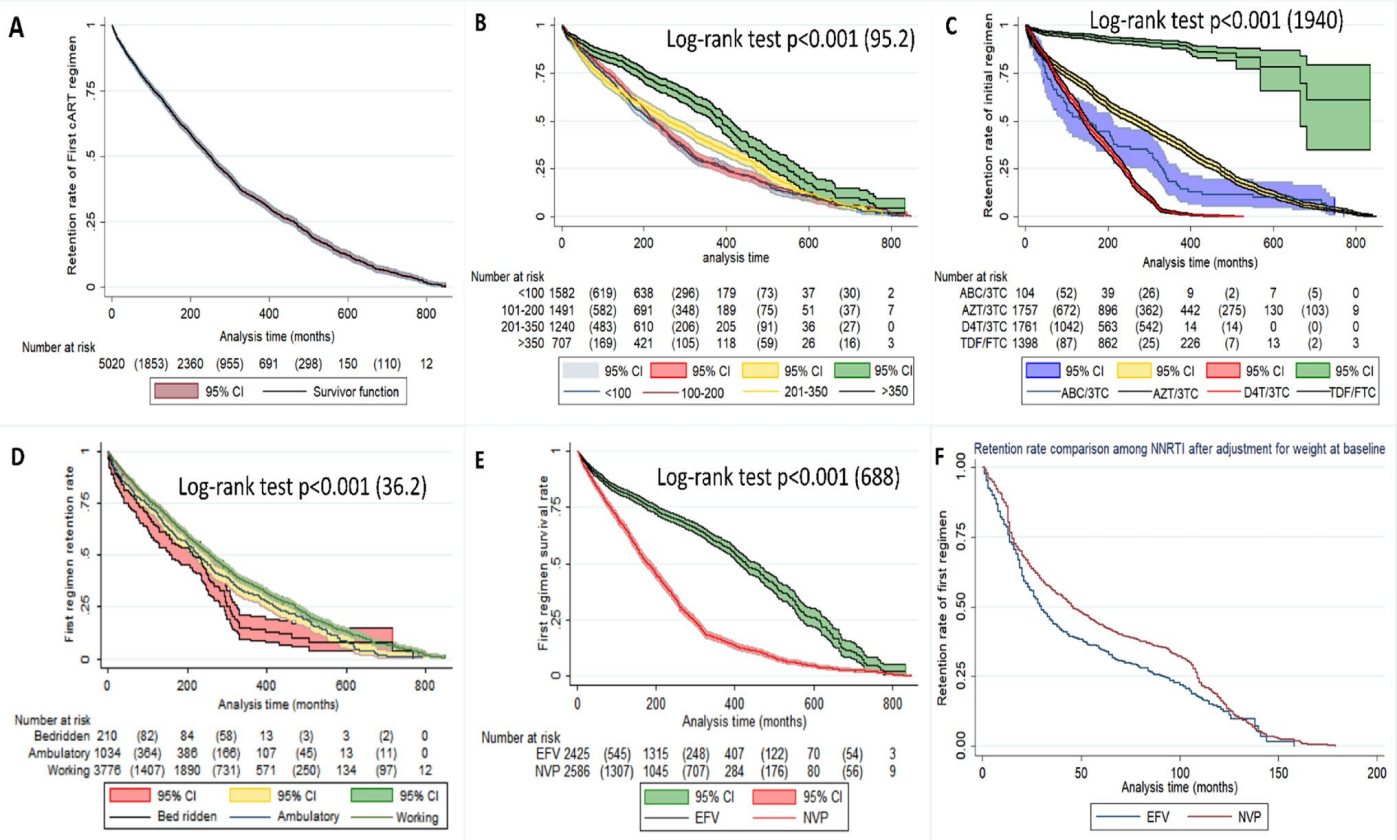
Unadjusted and adjusted Kaplan–Meier curves for: overall retention rate of first regimen across the follow-up months (**A**), unadjusted difference of retention rates among CD4 + T cell counts (**B**), unadjusted difference of retention among the initially used cART backbones (**C**), unadjusted difference across categories of initial functional status (**D**), unadjusted difference of first regimen retention between the NNRTIs used (**E**) and figure F represents adjusted difference of NNRTI for initial body weight.

Table 3 presents mean survival duration and cART modification rates- first through fifth modification across different intervals of follow-up. In this analysis, the mean survival duration of modification was as follows: initial regimen, 59 (57–61); second regimen, 39 (36–50); third regimen, 50 (46–56); fourth regimen, 3 (3–4); and fifth regimen, 16 (15–17). On the other hand, modification rate of cART per 1000 PMFU 95% CI were 3 (95% CI 2.9–3.1); 10 (95% CI 9.5–10.5); 5.6 (95% CI; 5.1–6.1); 8.8 (95% CI 7.6–10.2); 10.5 (95% CI 7.9–13.3) for initial, second, third, fourth and fifth regimen, respectively. Furthermore, Kaplan–Meier curves for cumulative survival on first cART regimen were plotted on specific variable subgroups (see Fig. 6 for details).

**Table 3:** Modifications (switches and substitutions) rate across the order of cART regimens used among PLWHA in hospitals of Asmara, 2005–2021.

Modification rate of cART regimens per 1000 person months (95% CI)					
Duration intervals (months)	Initial regimen	Second regimen	Third regimen	Fourth regimen	Fifth regimen
Overall	3 (2.9–3.1)	10 (9.5–10.5)	5.6 (5.1–6.1)	8.8 (7.6–10.2)	10.5 (7.9–13.3)
0–6	3.7 (3.1–4.5)	8.5 (7.2–10)	7.6 (6–9.6)	12.8 (9.1–17.9)	15.1 (8.9–25.5)
6–12	3.5 (2.8–4.2)	9.9 (8.5–11.6)	6.4 (4.9–8.5)	3.1 (1.4–6.5)	6.6 (2.7–15.9)
12–24	2.7 (2.3–3.2)	17.2 (15.6–18.9)	6.3 (5.1–7.8)	9 (6.3–12.8)	9.2 (4.6–18.5)
24–36	2.8 (2.4–3.3)	9.1 (7.9–10.6)	6.1 (4.8–7.8)	10.3 (7.1–14.9)	17.4 (9–33.5)
36–48	2.4 (2–2.8)	7.3 (6.1–8.6)	6 (4.7–7.8)	10.5 (7–15.8)	4.4 (1.1–17.8)
48–60	2.3 (1.9–2.7)	6.4 (5.2–7.8)	3.5 (2.5–5)	7.1 (4.1–12.3)	8.4 (2.7–26.3)
60–72	2.8 (2.3–3.3)	6 (4.8–7.4)	2.9 (2–4.4)	4.8 (2.4–9.7)	14.9 (5.6–39.7)
72–84	2.4 (2–3)	3.7 (2.7–5)	4.1 (2.9–5.9)	12 (7.4–19.7)	4.6 (0.6–32.8)
84–96	2.1 (1.7–2.6)	6.2 (4.9–8)	6.6 (4.9–9)	10.7 (5.9–19.4)	12.5 (3.1–49.9)
> 96	3.2 (3–3.3)	20.1 (17.8–22.7)	5.6 (3.9–8)	5.5 (2.3–13.4)	0
Median (95% CI), months	59 (57–61)	39 (36–50)	50 (46–56)	3 (3–4)	16 (15–17)

### Cox proportional Hazards model for independent predictors of cART modification

In the adjusted multi-variable Cox-proportional hazards model, likelihood of cART modification was associated with Organisation (Hospital B: aHR = 1.293, 95% CI 1.162–1.439, *p* value < 0.001) (Hospital D: aHR = 1.799, 95% CI 1.571–2.060, *p* value < 0.001) with respect Hospital A (ref.); Initial weight (aHR = 0.996, 95% CI 0.993–0.999, *p* value = 0.013); address (Inside Maekel: aHR = 0.854, 95% CI 0.774–0.942, *p* value = 0.002); Initial WHO clinical stage (Stage III :aHR = 1.116, 95% CI 1.116–1.220, *p* value < 0.001) with WHO clinical stage I (ref.); NRTI (AZT-based: aHR = 0.654, 95% CI 0.515–0.830, *p* value < 0.001) (d4T Based: aHR = 1.849, 95% CI 1.449–2.360, *p* value < 0.001) (TDF Based: aHR = 0.068, 95% CI 0.051–0.091, *p* value < 0.001) with respect to ABC (ref.) and NNRTI (NVP Based: aHR = 0.889, 95% CI 0.806–0.980, *p* value = 0.018). See Table 4.

**Table 4:** Cox-proportional hazards for factors associated with modification of initial cART regimen among PLWHA in hospitals of Asmara, 2005–2021.

Cohort characteristics	Unadjusted Hazards ratio		Adjusted Hazard Ratio	
	aHR	*p* value	aHR	*p* value
**Organization**				
A	1		1	
B	0.975 (0.882–1.077)	0.615	1.293 (1.162–1.439)	**< 0.001**
C	0.806 (0.734–0.885)	**< 0.001**	1.058 (0.949–1.179)	0.310
D	0.865 (0.769–0.973)	**0.016**	1.799 (1.571–2.060)	**< 0.001**
**Age**	1.009 (1.006–1.012)	**< 0.001**	–	–
**Initial weight**	0.995 (0.992–0.998)	**< 0.001**	0.996 (0.993–0.999)	**0.013**
**Initial functional status**				
Bed ridden	1		–	–
Ambulatory	0.779 (0.650–0.935)	**0.007**	–	–
Work	0.660 (0.558–0.781)	**< 0.001**	–	–
**Address**				
Outside Maekel	1	**0.021**	1	**0.002**
Inside Maekel	1.122 (1.018–1.237)		0.854 (0.774–0.942)	
**Initial clinical stage**				
Stage I	1		1	
Stage II	1.137 (1.026–1.259)	**0.014**	0.998 (0.900–1.108)	0.977
Stage III	1.250 (1.148–1.360)	**< 0.001**	1.116 (1.021–1.220)	**0.015**
Stage IV	1.292 (1.135–1.470)	**< 0.001**	1.114 (0.969–1.280)	0.129
**NRTI**				
ABC-based	1		1	
AZT-based	0.696 (0.559–0.867)	**0.001**	0.654 (0.515–0.830)	**< 0.001**
D4T-based	1.826 (1.463–2.278)	**< 0.001**	1.849 (1.449–2.360)	**< 0.001**
TDF-based	0.086 (0.065–0.114)	**< 0.001**	0.068 (0.051–0.091)	**< 0.001**
**NNRTI**				
EFV-based	1	**< 0.001**	1	**0.018**
NVP-based	2.643 (2.450–2.851)		0.889 (0.806–0.980)	

Significant values are in bold.

TDF, Tenofovir disoproxil fumarate; FTC, Emtricitabine; AZT, Zidovudine (AZT); 3TC, Lamivudine; ABC, Abacavir; D4T, Stavudine; NVP, Nevirapine; EFV, Efavirenz. NNRTI, non-nucleoside reverse transcriptase inhibitor; NRTI, nucleoside reverse transcriptase inhibitor.

## Discussion

This study pooled data of four cohorts of patients enrolled in cART programs in Asmara, Eritrea from 2005 to 2021. Overall, we observed a high cumulative frequency of cART modification, 3223(64%) comprising of substitutions, 2956(58.8%); switching, 37 (0.7%); and both, 230 (4.5%). This translated into an all-cause modification rate of 12.3 (95% CI 11.9–12.8) per 1000 PMFU (Substitution rate of 12.3 (95% CI 11.9–12.8) per 1000 PMFU) and Switching, 3.9 (95% CI 3.2–4.8) per 10,000 PMFU). Although data for comparisons are limited due to heterogeneity of designs, proximity (*post facto*) to WHO cART treatment guidelines revisions, access to routine HIV RNA-VL testing, and highly variable follow up period; the high proportions of substitution and the heavy tilt towards SDS involving NRTI and/or NNTRI or INSTIs is common in SSA. In a study in Nigeria (period: 2004–2006), up to 80% of patients enrolled in a tertiary cART care facility over a period of 8 years experienced regimen modification^6^. Further, a recent systematic and meta-analytical review from Ethiopia reported that the pooled magnitude of regimen modification was 37% (95% CI 34–44%)^11^. In Côte d'Ivoire (period: 2004–2006), 483 (24%) patients experienced treatment modification at a rate of 20.7/100 patient-years (PY) (95% CI 18.9–22.7)^12^. Studies in Kenya, Uganda, Ethiopia, and West Africa reported lower levels of modifications rates: 18.6/100 PY, mmm, 10.1/100 PY, 16.2/100 PY, respectively^8,13–15^.

In general, treatment guidelines for cART suggest that regimens should be individualized and that patients should have some degree of autonomy. In this process, virological efficacy, possible AEs, DDIs, childbearing potential, among others should be considered^16^. However, data in most treatment programs in SSA points at the fact that individualization of regimens is overly complicated by the limited cART backbone options. Consequently, substitutions due to patients’ and physicians’ decisions are limited and a large number of substitutions are based on non-clinical considerations. In most situations, a disproportionate number of substitutions are triggered by programmatic guideline changes and subsequent substitutions prioritize adherence to extant treatment guidelines. Furthermore, choices are limited to available stock^6^. These patterns are clearly demonstrated in Fig. 3. It is clear from this illustration that the high substitution, hence modification rates, observed after 2010 were largely due to the phasing out of d4T based regimens as per WHO 2010 directives. In a 2010 report, the WHO consolidated guideline recommended replacement of d4T with TDF^17^. The recommendation was informed by high value evidence connecting d4T to lactic acidosis (LA), peripheral neuropathy, lipodystrophy, and AEs associated with mitochondrial toxicity^18^. In prior years, patients in these settings were retained on d4T (despite active toxicity and the absence of routine viral load (VL) data) for a much longer duration, 59 (IQR 57–61) months. At the very least, our results underscore the importance of considering the content and revisions of treatment guidelines when evaluating long-term drug—modification rates in cART programs in SSA.

Many studies have reported toxicity/intolerance as a major driver of cART modification globally^8,11,19–21^. In Ethiopia, pooled data from 17 studies identified intolerance/toxicity as the major cause of treatment modification (8% (95% CI 46, 69%; Range: 14.4–88.5%)^11^. Additional factors included TB co-morbidity (58% (95% CI 46—69%), treatment failure (7% (95% CI 5–9%), and pregnancy (5% (95% CI 4–7%). With the exception of toxicity as the leading cause of cART modification, a different pattern was observed in this study. Overtime, the frequency of toxicity as a leading cause of cART modification decreased. A possible explanation of this outcome is the fact that TDF-based (and lately DTG-based regimens) are less toxic compared to d4T or AZT-based regimens. This claim is supported by multiple reports from the region^19,22^. Further, distribution of toxicities reflected the established AEs of NRTI and NNRTIs (lipodystrophy, anemia, peripheral neuropathy, and skin rash, among others). Importantly, our data suggests that the phasing out of d4T-based regimens (d4T/3TC/EFV or d4T/3TC/NVP) and the limited use of AZT-based regimens (AZT/3TC/EFV or AZT/3TC/NVP) or PIs has not diminished the burden of lipodystrophy in patients on cART. The importance of this issue is likely to increase further in coming years with the ongoing transition to DTG-based regimens.

While toxicities associated with specific regimens were reported; we should note that the problem was not documented in full. This can largely be attributed to the lack of necessary laboratory infrastructure. Reflecting on this problem, Castelnuovo et al., argued that the low of modification of TDF-based cART (used widely in these programs) may not reflect the magnitude of TDF-related toxicity in many programs in LMICs. In their opinion, laboratory evaluation of toxicity is rarely supported by national programs due to the high cost of relevant diagnostic technologies—clinical chemistry analyzers or imaging equipments such as X-ray absorptiometry (DEXA)^13^. By embracing this argument, we can assert that its remains unclear whether AEs requiring specific laboratory measurements (Lactic acidosis, creatinine clearance (CrCl), loss of bone mineral density (BMD), dyslipidemias) were present in this population. As such the extent to which these abnormalities may have influenced ARV modifications remains unclear.

In cART modifications studies, it has been suggested that substitutions-related to cART shortages negates much of the benefit sought by cART program implementers^23^. Stock outs of testing kits and ARVs can prompt unnecessary delays in cART initiation and trigger unstructured treatment discontinuations or interruptions^24^. At the program level, it amounts to inefficient use of scarce treatment resources. Drug stock outs can also elevate the risk of OIs, TF, viral resistance and death^25,26^. Therefore, drug-shortage related substitutions observed in this setting point at a significant programmatic gap. The importance of this problem is magnified by the observed connection between rising number of substitutions and rising frequency of shortage-related substitutions. Although the pattern of stock out-triggered substitutions observed in this setting may be unique, stock outs are common in cART programs in SSA. A study in Kinshasa reported TDF/3TC/EFV stock outs in a large number of high burden facilities^27^. In South Africa, policy shift towards the use of TDF-based regimens as the preferred first line treatment was associated with stock outs^27^. By and large, reports from the regions have partly attributed this problem to the inability to adjust cART supply to existing/or potential demand. Others have linked the problem to insufficient human resources and poor infrastructure. Whether, these influences are at play in this setting is hard to discern. Regardless, research on the consequences of stock-out triggered substitutions in cART programs in the region is urgently needed to inform the ongoing DTG-based treatment scale-ups.

In our analysis, regimen switches due to treatment failure were low, 37 (0.75) and 230 (4.5%) for both. The low switch rate is a common feature in SSA. For example, a large-scale multi-country cohort analysis reported a low switching rate of 1.63/100 [95% CI 1.60–1.66] PY observation^28^. Interestingly, relatively high switch rates and low substitution rates have been reported in some high income countries (HICs). Attempts to explain this LMICs-HICs disparity in switching has raised multiple possibilities. Some have argued that treatment programs in SSA lack proper mechanisms to identify treatment failure (TF) and that limited alternative treatment options do not allow a switch^5,28^.

To support this assertion, they aver that the lack of infrastructure including plasma HIV RNA-VL assays and standard genotypic resistance testing limits the ability to detect TF. Interestingly, a multi-country study involving 300,000 HIV-positive patients noted that rate of switching is largely determined by monitoring strategies—HIV RNA-VL, CD4^+^ T-cell count and clinical presentations. In the absence of routine HIV RNA-VL monitoring, switching occurred later, and at lower CD4^+^ T-cell counts^28,29^. Confirming this assumption, Haas et al. reported switch rates of 3.15/100 (95% CI 2.92—3.40) PY for programs with routine HIV RNA-VL monitoring, 1.21/100 (1.13–1.30) PY for programs with targeted HIV RNA-VL monitoring, and 0.49/100 (0.43–0.56) PY for programs with clinical monitoring. Additionally, some studies have shown that switching following detection of TF can also be delayed due to lack of cART options^27^. Much of what is detailed in these descriptions applies to treatment programs in Eritrea. Although existing guideline recommend routine VL monitoring, targeted monitoring which depend largely of physician’s discretion and availability of HIV RNA-VL testing reagents is more common. A potential consequence of this approach includes long lag-time between diagnoses of TF and switching^30,31^. All in all, improved understanding of the rates of VF, possible lag-time between detection of HIV RNA-VL and switching, and its overall impact on patient’s outcomes are warranted.

In the univariate analysis of factors associated with initial cART modification, substitutions rates differed across a range of factors including hospital of care, baseline weight, address, initial WHO clinical Stage, initial CD4^+^ T-cell count, initial functional status, and NRTI or NNRTI used. In the multivariate Cox regression analysis, independent predictors of initial cART modification included residence outside Maekel; late initial WHO clinical stage, certain NRTI and NNRTI. In the adjusted multivariate Cox regression analysis, higher hazards of cART modification were associated with organization, unit reduction in initial weight, residence inside Maekel, initial WHO stage (Stage III), NRTI and NNRTI used. Most of these associations are not unique^11,14^. In their report, Njuguna et al. reported the low incidence of drug substitution for TDF-based regimens (2.6 per 100 P/Ys), compared to AZT and d4T based regimens (8.5 per 100 P/Ys vs. 17.9 per 100 P/Ys)^32^. Separately, substitution rates of 27.0/100 PYs; 1.9/100 PYs; 2.0/100 PYs were recorded for d4T, AZT and TDF-based regimens in a study in Kenya^22^. Many have suggested that these results highlight the better safety profile of TDF-based backbones^22^. Similarly, low CD4^+^ T-cell count has been described as an important predictor of cART modification^11,33^. According to some accounts, patients initiating cART at higher CD4^+^ T-cell count or with WHO Stage I disease are less likely to suffer from AIDS-defining illnesses that require treatment with drugs that may interact adversely with active cART regimens. A similar argument can be applied to the observed link between initial functional status and baseline weight and rate of cART modification. However, the link between address or Organisation and substitution rates remains unclear. This notwithstanding, it is clear that differences in Organization points at quality problems in particular institutions and the need for standardization.

Lastly, prior studies have reported conflicting findings on whether NVP-based anchors compared to EFV-based anchors are associated with higher modification hazards. Inzaule et al.reported an incidence rates of 9.80 (95% CI 5.28–18.22) for EFV vs. 7.17 (95% CI 5.58–9.21) for NVP^8^. In practice, however, they noted that the rate of cART modification due to toxicity/intolerance was higher with NVP as compared to EFV^8^. In this study, Kaplan–Meier analysis of initial regimens demonstrated a higher substitution rate for NVP-based regimes (6.7/1000 (6.3–7.2) PM for EFV-Based regimen vs. 18.6/1000 (17.8–19.4) PM for NVP-Based regimen. However, after adjusting for weight at baseline, NVP-based regimens demonstrated a more favorable retention rate. In the unadjusted Cox regression analysis, NVP-based regimens had a 2.64 (2.45–2.85) hazard of cART modifications, *p* value < 0.001. In contrast, the adjusted model demonstrated a lower hazard, 0.89(0.81–0.98), *p* value = 0.018. The likely explanation for this finding is that patients with high baseline weight have better tolerance for NVP-based regimens.

### Strengths and limitations

This study has multiple strengths and limitations. First, the study has a robust data set complete with detailed person-level clinical and demographic data. Secondly, few studies are able to capture long-term treatment modification patterns. In most part, analysis is rarely extended beyond initial regimen and reasons for modifications of partially detailed. In this study, we extended the analysis from the first to the fifth modifications. Consequently, the study provides a more comprehensive picture of cART modification history in four of the largest treatment centers in Eritrea. These strengths notwithstanding, our study has a number of limitations. These include the limitations associated with retrospective studies—missing data problems and non-standardized recordings of key variables.

## Conclusion

In conclusion, we report the first data describing cART modification and associated factors in Asmara, Eritrea. According to our analysis, most patients have been on NRTI and/or NNRTI. Use of PIs or INSTIs is still limited. The cumulative frequency of all cause first-line cART modification was 3223 (64%). Substitutions were disproportionately higher than switching suggesting possible deficiencies in HIV RNA-VL monitoring. Furthermore, toxicity/intolerance was a leading cause of initial cART modification. However, the impact of toxicity as a driver of cART modification has waned. From the second cART modification onwards, drug shortage was the predominant reason for change. The clinical significance of the high number of shortage-related substitutions at patient level is currently unknown. However, we believe that the outcome points at a significant programmatic gap. In the adjusted multivariate Cox regression analysis, higher hazards of cART modification were associated with treating hospital, unit reduction in initial weight, residence inside Maekel, initial WHO stage, NRTI and NNRTI used.

## Data Availability

The dataset supporting the conclusions of this article is available from the corresponding author on reasonable request.

## Abbreviations

3TC: Lamivudine
ABC: Abacavir
AZT: Zidovudine
d4T: Stavudine
DTG: Dolutegravir Dolutagravir
FTC: Emtricitabine
cART: Combined antiretroviral therapy
EFV: Efavirenz
INSTIs: Integrase strand inhibitors
LMIC: Low-middle income countries
NRTI: Nucleoside reverse transcriptase inhibitors
NNRTI: Non-nucleoside reverse transcriptase inhibitors
NVP: Nevirapine
OI: Opportunistic infections
PI: Protease inhibitors
PMFU: Person-months follow up
PTB: Tuberculosis
PY: Person-years
RNA-VL: Ribonucleic acid viral load
SSA: Sub-Saharan Africa
TDF: Tenofovir disoproxil fumarate
TF: Therapy failure
VF: Virologic failure
WHO: World Health Organization
